# Context of data sharing practices in collaborative human genomic research in low and middle income countries: A systematic review

**DOI:** 10.1371/journal.pone.0354471

**Published:** 2026-08-07

**Authors:** Deborah Ekusai-Sebatta, Moses Ocan, Shenuka Singh, David Kyaddondo, Dickens Akena, Alison Annet Kinengyere, Eve Namisango, Ekwaro A. Obuku, Erisa Mwaka

**Affiliations:** 1 Makerere University College of Health Sciences, School of Biomedical Sciences, Department of Anatomy, Kampala, Uganda; 2 Makerere University College of Health Sciences, School of Biomedical Sciences, Department of Pharmacology, Kampala, Uganda; 3 Africa Centre for Systematic Reviews and Knowledge Translation, Kampala, Uganda; 4 University of KwaZulu Natal, Discipline of Dentistry, Durban, South Africa; 5 Makerere University College of Health Sciences, School of Medicine, Child Health and Development Centre, Kampala, Uganda; 6 Makerere University College of Health Sciences, School of Medicine Department of Psychiatry, Kampala, Uganda; 7 Cicely Saunders Institute of Palliative Care, Policy and Rehabilitation Florence Nightingale Faculty of Nursing, Midwifery and Palliative Care, Kings College London University of London, London, United Kingdom; 8 Makerere University College of Health Sciences, School of Medicine, Department of Medicine, Clinical Epidemiology Unit, Kampala, Uganda; ^9^ London School of Hygiene and Tropical Medicine, Faculty of Epidemiology and Population Health, Kampala, Uganda; The World Bank Group, CANADA

## Abstract

**Background:**

The collection and aggregation of individual genomic data into large-scale repositories is now a common approach in biomedical research. Funding agencies increasingly require researchers to include data sharing plans in new project proposals, unless there are strong, clearly justified reasons. While sharing human genomic data promotes scientific discovery, innovation, and transparency, it also raises significant ethical, legal, and social concerns (ELSI). This review collated evidence on data sharing practices, context, facilitators and barriers in collaborative human genomic research in low and middle income countries (LMICs).

**Methods:**

The systematic review was done following *a priori* criteria. A protocol was registered in PROSPERO (CRD42022297984) and published with PLOS ONE journal. The articles were imported into EndNote software, duplicates were removed and the remaining articles were then transferred to Epi-Reviewer software. Independent reviewers (DES, LN; GK, DES) screened the articles for inclusion and extracted data in pairs. Any disagreements between the reviewers were resolved through discussion and consensus. The JBI checklist was used for assessing quality of the included articles and studies were classified as good, fair or poor. The assessment yielded overall ratings of good which demonstrated sound methodological rigor. We did not exclude any study from our analysis. Seven distinct categories emerged from the narrative synthesis.

**Results:**

A total of 2061 articles were identified from the initial search (PubMed, 594; Web of Science 340; Google scholar, 1127; and 30 from Bibliography search). The review included 11 articles and explored the context and the ELSI of sharing genomic data. The results included the practice of sharing data collaboratively, the ethical issues identified included: informed consent, data misuse and mistrust, inequity, the social dimensions included stigma and discrimination and the legal issues include data ownership and data protection. The barriers included mistrust and inequity in collaborative research and over regulation.

**Conclusion:**

Overall, trust and comprehensive cultural consenting process are critical during data sharing. Emphasis should be placed on striking a balance between protecting rights of research participants, the interests of researchers from LMICs and promoting scientific research. Policymakers should establish ethical and regulatory frameworks that emphasize equity and fairness in collaborative relationships.

## Introduction

### The context of data sharing in genomics research

Data sharing is the practice of making research outputs available to other researchers by adding or combining research participants’ data into larger repositories [1]. The nuances between genomic data sharing and transfer include: data sharing involves making genomic information, such as DNA sequences, available to a wider scientific community to promote collaboration and scientific discovery. It also refers to making data accessible for use by others beyond the research team, often through controlled or open-access repositories, collaborative platforms, or federated systems. Data sharing emphasizes enabling research, reproducibility, and collaboration while considering privacy, consent, and governance frameworks. In contrast, data transfer refers to the act of moving genomic data from one location to another, such as between servers, institutions, or individuals, with the goal of facilitating data for analysis or interpretation [2]. In addition, data transfer entails the point-to-point movement of data which could be the physical or digital movement of data from one entity or jurisdiction to another. Formal agreements and adherence to data protection laws may be required to enable this process, which is often subject to legal and regulatory scrutiny particularly in cross-border contexts. Such agreements typically define the parties involved, the specific systems, and the operational purposes (for example, data storage, processing, or analysis) without granting broader access rights. An increasing number of government departments, research communities, funding agencies and scholarly journals are developing initiatives and policies to promote data sharing and greater access to data, recognizing their enormous potential for scientific, social, and economic growth [3–6]. Open data policies from European countries [7,8] and the United states of America Wheeland [9] increasingly require custodians of human genomic data to make it as widely available as feasible [10]. Data sharing is regarded as essential for enabling and promoting genomic research in a way that will maximize the benefits to public health [11]. Additionally, several frameworks guiding the sharing of genomic data have been developed some of which are: the Genomic Data Sharing Policy [12], International Declaration on Human Genetic Data [13], International code of conduct for genomic and health related data sharing [14] and the Framework for Responsible Sharing of Genomic and Health Related Data [15]. The frameworks indicate that the rights of participants and their communities should be protected, they emphasize the importance of informed consent, de-identification of the shared data, privacy and confidentiality, sharing benefits from the study with the community where the participants were recruited from and ensuring everyone has access to the shared data, placing mechanisms in place to ensure that the shared data is not used to discriminate and stigmatize the individuals. They further indicate that transparency and trust are key components in the sharing of genomic data.

Genomic research is increasingly becoming common with researchers participating in different projects required to share details of their participant data. The sharing of genomic data promises to increase research efficiency, expedite translation of research results, and ensure the traceability and transparency of published studies and maximize the utility of results [16–19]. Research funders and sponsors demand that genomic sequences be deposited on public data repositories unless there are justifiable reasons why this should not be so [20]. The need for broad access to genomic data brings along a host of ethical concerns, including those related to privacy, confidentiality as well as fairness and equity [21]. The risk of re-identifiability remains a major ethical concern in genomic data sharing, even with the implementation of technical safeguards such as controlled-access repositories, encryption, pseudonymisation, and secure data transfer protocols [22–24]. Challenges of effective data sharing include: 1) absence of established standards for data users, 2) researchers from low and middle income countries (LMICs) often experience inequities in collaborative research, including not being appropriately credited for their contributions, 3) loss of intellectual property rights, 4) misuse of data [25,26] and 5) absence of benefit sharing frameworks [27,28]. These rooted disparities highlight the need to decolonize research ethics by promoting fair authorship practices, shared governance, and more equitable participation in global health research [29,30]. An analysis of genomic guidelines, policies, and procedures from LMICs by Ali et al 2021 [31] and de Vries [32] revealed significant weaknesses and gaps in the governance of genomic research and biobanking [31,33]. Several LMICs have enacted legislation through Data Protection Acts to protect personal data, however these laws often lack sector-specific provisions and detailed guidance for health research, presenting a significant challenge [34]. Most sub-Saharan countries lack ethical and legal frameworks to guide and regulate the sharing of genomic research data. The shared genomic data is inclusive of both genotypic and phenotypic data. Discussions should be held with researchers over whether data providers should review results before publication, collaborate on the analysis, approve the analysis plan in advance which are important areas for dialogue on data sharing [26].

Although data sharing enhances efficiency and holds great promise for advancing scientific knowledge and improving public health outcomes, it also presents a range of complex ethical, legal, and social issues (ELSI). These challenges include concerns around privacy, informed consent, data ownership, equitable access, and the potential for misuse or discrimination, all of which are especially significant in the context of genomic research [21,35–40]. One of the key challenges is determining how to protect the privacy of participants while enabling the sharing of data through global research networks [41]. Several concerns regarding privacy, discrimination, data misuse, inequity and stigmatization among many have led to policy responses from the National Human Genome Research Institute and additional policies from domestic and international countries that reaffirmed the recommendations for publicly sharing genomic data [17]. These policies restrict access and occasionally limit the utility of data to genomic data as a means of protecting research participants [17]. Only bona fide individuals with appropriate institutional credentials are granted access to permissioned data for ethically approved purposes under this policy. To promote ethical and equitable data sharing in genomic research, there is need for appropriate regulatory frameworks. However, the development of such frameworks should take into consideration the perspectives of the various stakeholders in the genomic research landscape to ensure that they are locally appropriate and context-specific.

Despite the valuable contributions made by earlier reviews, they remain limited in scope. Many of them focus on African or high- income settings and primarily examine policies, guidelines, and public attitudes toward genomic data sharing, with some restricted to pediatric contexts. For instance, a review by Ali et al. [31], mapped ethics-related guidelines and legal instruments governing genetic and genomic research across African countries, providing a policy-level overview rather than an analysis of data-sharing practices [31]. Rahimzadeh, Knoppers, and Bartlett [42] reviewed ethical, legal and social issues related to sharing whole-genome data, but their focus was restricted to pediatric genomics, thereby excluding broader stakeholder and institutional perspectives. More recently, Amoakoh-Coleman et al. [43] described ethical considerations for biobanking and genomic data use in Africa; however, their emphasis remained on biobank establishment, consent frameworks and regulatory gaps, without examining how data are shared in practice or how contextual factors influence data access, reuse or governance [43]. A review by Shabani Identified key concerns influencing willingness to share data, but did not examine how these attitudes interact with governance systems, but did not examine how these attitudes interact with governance systems, institutional practices or consent implementation in real settings, particularly in LMIC collaborations [44].

This review collated evidence on data sharing practices, context, facilitators and barriers in collaborative human genomic research in LMICs. Moreover, the review is justifiable because it was conducted to inform ethical data or guide collaborative human genomic research and it will enhance the body of evidence on ethical, legal and social implications of data sharing in collaborative genomic research setting criteria for data sharing. This review broadens the scope by examining genomic data sharing in Africa, Asia and other LMICs, alongside collaborations with the UK, US, Canada and Europe, allowing comparison of governance challenges in both Global North–South and South–South partnerships.

## Materials and methods

### Protocol development

A protocol was developed according to the Preferred Reporting Items for Systematic Reviews and Meta-Analyses (PRISMA) checklist recommended for systematic reviews [45–48]. This protocol was further developed and subsequently published in PLoS ONE [49].

### Definition of LMICs

In this review, LMICs are defined according to the World Bank’s 2024 country income classification, which categorizes economies based on gross national income (GNI) per capita. Countries with a GNI per capita below US$13,846 were considered LMICs [50,51]. While a substantial proportion of the included studies were conducted in African countries, the review also incorporates research from Asia and other LMIC regions.

### Review question

What is the context of sharing human genomic data in collaborative research in LMICs between 2003–2025?

The elements of the population, intervention, context, outcomes, setting and timeframe (PICOST) are presented in Table 1.

**Table 1:** Elements of PICOST.

Element	Description
Population	Researchers, research participants (children and adults), regulators and funders
Setting	Articles on data sharing in genomic research work in LMICs
Intervention/exposure	Sharing raw data sets, intermediate data and analyzed data
Context	Global North–South and or South–South collaborative research projects
Outcomes	Primary outcome: Data sharing Secondary qualitative outcomes: context, (types of data shared, how it is shared) data sharing practices, perceptions on data sharing, perceived barriers and facilitators to data sharing, ethical social and legal implications, pros and cons of data sharing, collaboration
Study designs	Quantitative: Cross sectional surveys, case control, longitudinal study, cohort Qualitative: Descriptive studies, narrative synthesis, case studies, ethnography, grounded theory Mixed methods: Both qualitative and quantitative study designs
Time period	2003-2025 (The year 2003 was chosen because lessons learned from the human genome project showed the importance of collaboration in genomic research)

### Time justification

The review focused on research conducted from 2003 onward, marking the completion of the Human Genome Project [41,52]. This period was selected because the project highlighted key lessons such as the importance of collaboration, data sharing, and the ethical management of genomic information that have since shaped how genomic research is designed and conducted.

### Eligibility and selection of papers

#### Screening of articles for inclusion.

All primary qualitative, quantitative and mixed methods research designs were eligible for inclusion.

The articles were retrieved from database searches and exported into EndNote software for initial screening [47]. Duplicates were removed through a two-step process: initial automated detection in EndNote followed by additional identification using EPPI-Reviewer, with all duplicates manually verified before removal. After de-duplication, all articles were screened by title and abstract.

### Data sources

The literature review involved a search of three electronic databases, namely PubMed, Google Scholar, and Web of Science in line with Cochrane Handbook recommendations to search at least two to three databases for comprehensive coverage [53–55].

### Inclusion criteria

All articles that met the PICOST criteria (Table 1), as defined by the research question, were included and constituted the unit of analysis. In addition, articles were included if they focused on data sharing in genomics and reported original research; both published and grey literature were considered. Date restrictions were applied to the initial electronic search to include articles published between 2003 and 2025, with the search limited to publications and we used English terms for search. Studies were included if they involved research collaborations between institutions in LMICs and those in high-income countries (Global North–South partnerships) or among LMICs themselves (South–South collaborations).

### Exclusion criteria

Papers were excluded if they focused on personal health records, clinical results, letters, opinion papers and articles that only focused on biobanking and not data sharing. We excluded papers that did not report relevant outcomes on genomic data sharing and those that did not stratify results for LMICs [49].

### Data abstraction and coding

Data extraction was done using a pre-designed and piloted form in Microsoft office excel [56] and EPPI reviewer software by a pair of reviewers, acting independently to capture the following information from included articles: author, year of study, country/region, study design and relevant outcomes [57]. The lead reviewer (DES) with another member of the team conducted data extraction in duplicate for a proportion of papers to enhance quality of the process. To assess the consistency of data extraction, a subset of papers was independently reviewed by two team members. The EPPI-Reviewer report [58] using the coding reports indicated a 92% level of agreement. Any discrepancies were discussed and resolved through consensus, further enhancing the rigor and credibility of the synthesis. Results of the full text extraction were shared with the remaining review authors to validate them.

### Coding and synthesis

A narrative synthesis was used to integrate and interpret findings from multiple included studies [59]. This approach was chosen due to the diversity in study designs, settings, and outcomes, which made quantitative synthesis inappropriate. We were guided by the framework proposed by Popay et al. [59] to ensure a systematic and transparent process. Data synthesis was conducted using a structured narrative approach, enabling the systematic identification and interpretation of key patterns across the included studies [60]. Two reviewers (DES and a research assistant) independently read and re-read each study to become thoroughly familiar with the content and to identify recurring themes and insights. The synthesis primarily relied on textual analysis to summarize and explain findings. As part of the preliminary synthesis, the reviewers gathered and arranged the findings from each study in a way that made it easier to see commonalities, differences, or trends across the studies. Key data such as study aims, methodology, population, and outcomes were extracted into a matrix developed in Microsoft Excel. This matrix also included relevant quotes and summary statements to preserve the context and depth of each study.

### Handling of missing data

Variables that were desired but missing or not reported were denoted as not reported (‘NR’) and clarification was sought by contacting the study authors. Papers with essential data that remained unavailable due to author nonresponse were excluded, as the missing information was necessary to determine eligibility and ensure methodological rigour. Excluding these studies ensured that the final evidence base was complete, verifiable and suitable for a reliable synthesis.

### Quality assessment

A publication assessment of all the included studies was done following the Joanna Briggs Institute (JBI) guidance standardized critical appraisal tool for assessing methodological quality [61]. Based on the total scores, studies were classified as good (8–10), fair (5–7), or poor (0–4), following published guidance and prior reviews using similar thresholds, ensuring consistency and transparency in our assessment of methodological rigor. This scoring involved evaluating how rigorously the research was conducted, including data collection and analysis methods. It was done to ensure the included studies were credible, trustworthy, and reliable. To enhance transparency in the synthesis, the appraisal scores were used to guide the inclusion and interpretation of evidence. Studies rated as poor were excluded from the review to ensure that only credible and methodologically sound evidence informed the findings.

## Results

### PRISMA flow chart of data sharing in human genomics collaborative research

**Fig 1:**
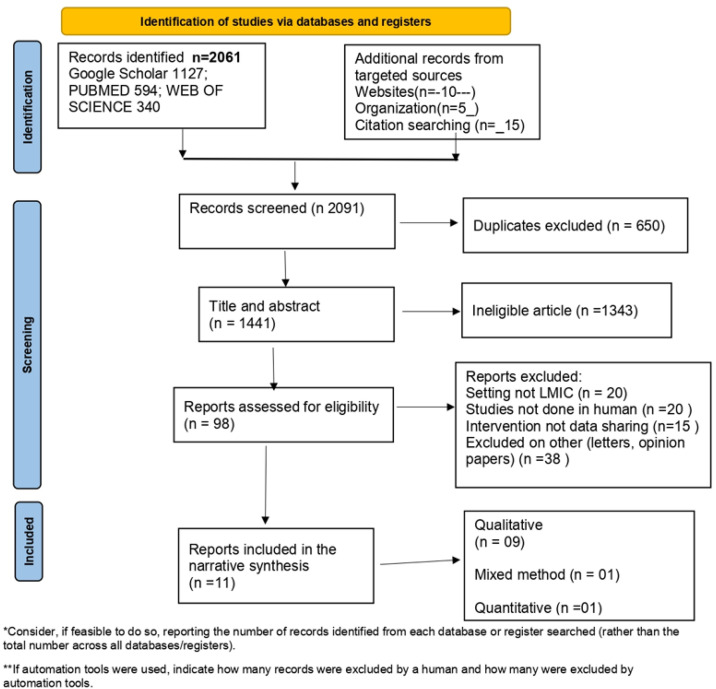
Showing the PRISMA flow chat.

A summary of the findings indicates that 2,091 records were identified through database searches: 1,127 from Google Scholar, 340 from Web of Science, 594 from PubMed, and 30 from targeted sources. After removing 650 duplicates, 1,441 records remained for title and abstract screening. Following full-text review of 98 articles, 88 were excluded for not meeting the inclusion criteria. Ultimately, 11 studies were included in the narrative synthesis (see Fig 1).

### Description of articles

A total of 11 studies were included in the systematic review of which nine were qualitative studies, one quantitative study and a mixed method study (Table 1). The qualitative articles included [32,62–69] and a mixed method article [70] and the quantitative article [25]. The studies focused on collaboration and data sharing in LMICs as well as on the sharing of human genomic data.

The characteristics of the included studies have been summarized in Table 2.

**Table 2:** Characteristics of included studies.

No	Author/year	Country of origin	Country where data collection was conducted	Design	Population	Sampling Criteria/sample	Data collection approach	Data collection methods	Nature of collaboration
1	de Vries [32]	South Africa	Kenya, Gambia and United Kingdom	Qualitative sociological study	Field workers Researchers REC members	Purposive sampling 49	Qualitative	Interviews and document analysis	Global North -South
2	Jackson [66]	Canada	Canada, the United States, and the United Kingdom and international organizations representing Global and LMICs	Phenomenology	Policy makers	Snowball sampling 22	Qualitative	interviews	Global North-South
3	Mafigiri [67]	Uganda	Uganda	Exploratory	Researchers	Purposive 15	Qualitative	Indepth interviews	Global South
4	Munung [68]	South Africa	Africa	Exploratory	Researchers	Purposive sampling 17	Qualitative	Interviews	Global South-North
5	Ekusai [65]	Uganda	Uganda	Phenomenology	Researchers REC members Regulators	Purposive 16	Qualitative	Interviews	Global South
6	Mweemba [71]	Zambia	Zambia	Case study approach	Researchers Members of RECs, lawyers and policy makers	Purposive 14	Qualitative	Interviews	Global South
7.	Cengiz [64]	South Africa	Sub Saharan Africa	Exploratory	REC members	Purposive through snow ball 20	Qualitative	Indepth interviews	Global South
8.	Brown [63]	South Africa	16 countries in sub Saharan Africa	Qualitative study design	Researchers	Purposive through snow ball 16	Qualitative	Indepth interviews	Global South-South
9.	Kabanda [70]	South Africa	43 countries	Descriptive cross sectional	Researchers	Purposive through snow ball 160	Mixed methods	Survey	Global South-South
10.	Anie [62]	United Kingdom	Ghana Nigeria Tanzania	Focus groups using qualitative methods	Patients parents/caregivers, community leaders, healthcare professionals	Purposive 112	Qualitative	Focus group discussions	Global North-South
11.	Bezuiden [25]	United Kingdom	13 countries in Sub Saharan Africa	Cross sectional design	Scientists	Purposive	Quantitative	Survey	Global South -North

A summary of the qualitative articles which were included in the review are summarized showing the themes and quotes (Table 3).

**Table 3:** Summary of the qualitative studies.

Domain	Summary of findings across studies	Illustrative studies	Representative insights or quotes
**1. Context of data sharing**	Genomic data sharing mainly occurred in collaborative projects (e.g., MalariaGen, biobanking initiatives). Data governance structures varied by country, with some relying on international guidelines. Local researchers often had limited awareness of long-term data use and control.	Mafigiri [67]; de Vries [32]; Brown [63]	“The consent was that we were going to work on malaria… that is sort of betraying the confidence that people give us.”
**2. Practice in data sharing**	Sharing practices were often informal or dependent on collaborations. Some institutions had data access committees, but enforcement and clarity on ownership were limited.	Kabanda [70]; Cengiz [64]	“Consumers of data are requested to sign non-disclosure agreements with confidentiality statements.”
**3. Ethical concerns**	Recurrent issues included informed consent, benefit sharing, and equitable access to data. Participants and researchers expressed fears of exploitation and data misuse.	Anie [62]; Ekusai [65]; Mweemba [71]	“People have always been afraid of people misusing especially genomic information.”
**4. Social dimensions**	Trust and mistrust were central themes. Mistrust stemmed from historical inequities, lack of feedback to communities, and limited involvement of LMIC researchers in decision-making.	Jackson [66]; Munung [68]; Ekusai [65]	“Countries simply do not understand the data they have… reacting out of pure mistrust.”
**5. Legal concerns**	Regulatory gaps existed around ownership, secondary use, and oversight. National guidelines often took precedence, but were inconsistently applied.	Mweemba [71]; Brown [63]; Cengiz [64]	“Before any publication… there should be authorization by the Ministry of Health.”
**6. Facilitators**	Facilitators included capacity strengthening, equitable collaborations, transparent consent, and local data governance structures.	Mafigiri [67]; Munung [68]	“Empower LMIC researchers to negotiate MTAs and data sharing agreements.”
**7. Barriers**	Key barriers were lack of infrastructure, weak regulation, inequitable power relations, and limited understanding of data sharing principles among stakeholders.	Ekusai [65]; Jackson [66]	“We do not have control once the data is sent out, especially to our people in the global North.”

### Publication assessment of the studies

We assessed the methodological quality of all 11 included studies in the systematic review. The assessment comprised qualitative, quantitative, and mixed-methods research, and the JBI appraisal checklist was used (see appendix 4, Table I and 4.1, Table 2) [61,68]. (Appendix 4, Table 1 for qualitative studies; Appendix 4.2, Table 2 for quantitative studies). The quality assessment yielded overall ratings of good indicating that the included studies demonstrated sound methodological rigor. None of them were rated as poor and because of this, we did not exclude any study from our analysis. Instead, we brought all the findings together, while keeping in mind the differences in how rigorous each study was when interpreting the evidence.

### Ethics approval and consent to participate

All empirical studies included in this review reported receiving the required ethical approval from their respective institutional review boards or ethics committees.

Additionally, this systematic review was approved by the Makerere University Higher degrees committee School of Biomedical Sciences Research ethics committee (SBSHD-REC 2022−273) and the Uganda National Council for Science and Technology (SS1730ES).

### Narrative synthesis

The narrative synthesis produced seven overarching domains, developed through iterative discussion and reflection to capture both frequent and salient findings. These domains were: 1) context of data sharing, 2) genomic data sharing practices, 3) ethical concerns, 4) social dimensions, 5) legal concerns, 6) facilitators, and 7) barriers.

#### 1. Context of data sharing.

The review of the 11 articles revealed that genomic data sharing was primarily conducted through collaborative relationships, either among LMICs or between LMICs and the Global North. Furthermore, trust and partnerships with clearly articulated collaborative objectives facilitated data sharing, in contrast to arrangements characterized by unclear communication. Two articles [65,68] indicated that the nature of the research collaborations between LMICs and high-income countries is often inequitable in terms of access and research funding. Regarding collaboration, four articles [62–64,70] involved working together with various stakeholders in sub Saharan Africa, including research institutions and community members, to ensure ethical data sharing practices. One article [64] indicated that cross boarder sharing presents unique challenges such as regulatory discrepancies which complicate the process of sharing data^.^

#### 2. Practices in the sharing of data.

Two articles [25,70] indicated the practices used in the sharing of data and some of those mentioned included publishing with peer reviewed journals and sharing through institutional repositories. Informal sharing practices mentioned were emailing data to friends and cloud services [70]. One article O Mweemba et al. [69] indicated strict regulation under the National Health Research Act. One article Anie et al. [62] indicated sharing data with the project collaborators as well as external scientists. One article Brown et al. [63] indicated the use of data management practices to ensure data quality and integrity. One article Munung et al. [68] indicated that externally funded projects have a played a critical role in building capacity of genomic research.

#### 3. Ethical implications.

*3.1 Fear of exploitation.* Three articles [65,67,68] highlighted fears of exploitation from the sharing of genomic data. The articles noted that while international health genomic partnerships are mutually beneficial, they allayed fears of exploitation through commercialization of the shared data. The fears were primarily shaped by past experiences of exploitation [67,68].^.^ Two studies reported on how the lack of standardized guidelines for benefit sharing places Africans at risk of exploitation [67]. Another article expressed concerns about the increasing desire to conduct research in Africa because of the human genomic diversity of Africans with either limited or no benefits to the African population [32].

*3.2 Informed consent.* Four articles [32,62,67,69] reported on the need for detailed informed consent and transparency and ensuring participants fully understand the genomics concepts. The need for comprehensive, culturally grounded consent processes anchored in honesty and transparency was emphasized. It was reported that benefit-sharing issues were rarely addressed in existing informed consent processes, and that challenges in obtaining informed consent for research in LMICs have been extensively documented [32]. One article highlighted that while some participants viewed the consent process as a means of protection, others feared that researchers might still engage in activities beyond what had been consented to and because of such fears specific consent was recommended [71].

*3.3 Risk of re-identifiability, breach of privacy and confidentiality.* Despite the sharing of de-identified genomic data, there remains a risk of re-identification, particularly when such data are deposited in public or semi-public databases where they can be cross-referenced with other data sources, such as demographic information. Genomic data are inherently identifying and cannot be fully anonymized or effectively de-identified, these practices heighten the risk of re-identification and raise concerns about potential stigma and discrimination affecting individuals and communities to whom the data relate.

Two articles [62,66] indicated that genomic sequences are routinely shared with patient level meta data, which may in some cases be directly linked to the genome. Such data-sharing practices may infringe on privacy and confidentiality. This is so because re-identifiability poses a significant risk when sharing genomic data because, even when such data is anonymized or de-identified, it can still be linked back to individuals whose data was shared. The re-identified data poses individuals and communities to privacy and confidentiality breach and risks which could lead to stigma and discrimination. Two articles [32,62] reported concerns for data protection of both privacy and confidentiality purposes and highlighted their importance during the sharing of genomic data.

*3.4 Commercialization and Benefit sharing.* One article highlighted concerns that profits could be generated from shared genomic data and that any resulting benefits may not be distributed equitably. There was also fear that if the data were uploaded to international databases, it might be used to develop new diagnostics or drugs that would later be sold back to LMICs at high and potentially unaffordable prices [66]^..^ Three articles [63,64,69] highlighted ethical concerns associated with the use of data for commercial purposes. The authors posited that genomic data on international databases could be used for the innovation of novel diagnostic and therapeutic interventions and the products sold to LMICs expensively. Three articles [65,67,68] attributed this state of affairs to the lack of benefit-sharing models in the current ethical and legal frameworks in LMICs.

Three articles [63,67,68]^.^reported that there should be fair and equitable sharing of the benefits of research with individuals and research communities. The authors noted that often it was not clear because the existing ethical and legal frameworks are vague on this issue.

*3.5 Data misuse.* Seven articles [32,62,63,65,66,69,70]^.^ indicated that shared genomic data are at risk of misuse because they are highly personal and can reveal sensitive information about an individual’s health.

Unauthorized access or sharing of this data can lead to privacy breaches, stigma and discrimination as well as have negative effects on the country where the data was from. Third-party companies may misuse shared genomic data for profit without the consent of individuals. Such misuse may include selling the data to other parties or using it to develop commercial products without compensating the individuals whose data contributed to the research.

*3.6 Inequity in the sharing of genomic data.* Three articles [65,67,68] articles highlighted inequity in the sharing of the data. It was indicated that data sharing resulted into disproportionate rewards for local scientists in LMICs where data was collected. They indicated that the often limited data analysis and writing skills meant local scientists who mainly contributed in generating most of the primary data collection gained the least from the outcomes of the research [67]. Another article indicated that it could be that African researchers as it has happened before are just being used for collecting materials because if you don’t have the capacity to analyze and make sense of the data then you collect and send it to people who can make sense of it [68].

*3.7 Power dynamics.* Two articles cited the role of power dynamics in the sharing of genomic data [25,65]. There is an imbalance in influence and decision-making power during collaborations, often favoring high income institutions [25]. This can disregard LMIC researchers and overlook their needs and perspectives in setting agendas and sharing data. They felt that power differentials put researchers from LMICS in a vulnerable position and heighten the risk of exploitation [65].

#### 4. Legal implications.

This section presents the legal issues identified from the articles, including data ownership, data protection, data sharing responsibilities, data access as well as the limited understanding of regulations governing genomic data sharing.

4.1 Ownership of the shared data. Two articles [67,69]^,^ reported the need to clarify ownership of shared and associated genomic data within national ethics guidelines.

One article [69] notes that the Zambian law notes that state specimens belong to the government and require governmental approval for publications.

Uncertainty remains about the rightful owner of the data and several researchers were unsure of the rightful owner and some advocated for the need for collaborative ownership of the shared genomic data.

4.2 Data protection. Five articles highlighted the importance of ensuring data protection and security of the shared genomic data [32,66–68,70]. The articles indicated that because genomic data can reveal not only personal health information but also familial relationships and predispositions to diseases. The need for data protection of shared genomic data is critical due to the sensitive and unique nature of genetic information. Without proper safeguards, the misuse or unauthorized access to this data can lead to privacy violations and discrimination.

4.3 Data sharing agreements. Two articles [32,64] indicated that researchers were required to develop mechanisms and policies for data release. Among the policies developed were data-sharing and transfer policies, which provided guidance on how data should be shared.

Data sharing agreements and standardized data transfer agreements are relevant because they clearly define these responsibilities which helps to protect the rights of participants, as well as maintain trust in research collaborations, and promote the responsible use of shared data. One article [67] indicated that researchers and institutions in LMICs should be empowered to negotiate data-sharing agreements, as this would enhance their ability to obtain adequate rewards from the research process. The article noted that collaborators from the Global South are at times unaware of the opportunities to negotiate more equitable and rewarding agreements.

Further, one article commented on the appropriateness and adequacy of the Zambian National Research Act on genomic research because the legislation seemed to promote Zambian domestic research capacity, including the country’s ability to store, process and use samples and data [71]. Concerns were raised because some of the statements in the Act seemed to be stifling research and genomics development in the country.

4.4 Lack of a regulatory framework. Two articles [63,70]^.^ cited the lack of frameworks particularly the absence of policies guiding the sharing of data were cited as barriers to sharing genomic data.

4.5 Limited understanding of the regulations governing genomic data sharing. One article [67], indicated that several local researchers were not conversant with the guidelines and regulations that govern genomic data sharing. Two articles [64,67] indicated that data sharing in international collaborations is oftentimes hindered by differences in regulatory frameworks across countries. They highlighted the need for harmonization of policies and regulations to facilitate data-sharing

4.6 Data access. Three articles [25,32,65] noted challenges related to access to shared data, particularly for researchers from LMICs. They reported that decisions about data access were often puzzling, as they were made by groups of researchers who were otherwise committed to an open-access agenda. The reasons cited for limited access included inadequate infrastructure, power disparities, digitalization and advances in data science, affordability constraints, and lack of awareness [65]^..^

#### 5. Social dimensions.

*5.1 Risk of stigma, labelling and discrimination.* Social implications included the risk of stigma, labelling and discrimination to individuals and communities in the event of a breach of confidentiality. Two articles contended that a data breach could lead to the re-identification of data and or sample of an already vulnerable population [32,66]. One article indicated that misuse or inadequate protection of sensitive data could pose risks of stigmatization and discrimination, potentially causing harm to the affected individuals or communities [64].

5.*2 Community engagement.* One article [63] highlighted the importance of involving communities in planning processes and emphasized the crucial role of community engagement in building trust. They noted that engaging communities through dialogue and culturally appropriate methods is vital for fostering trust.

#### 6 Facilitators to data sharing.

*6.1 Perceived benefits of data sharing.* Three articles reported that researchers perceived data sharing as having positive implications for research. These articles indicated that sharing data could advance research, increase visibility, and foster collaboration and more equitable research partnerships [25,63–65,67].

*6.2 Positive attitude towards sharing data.* Two articles [62, 70] indicated that willingness to share data was attributed to de-identification and the protection of privacy and confidentiality. One article further indicated that willingness to share data was linked to institutional capacity for data storage [70].

*6.3 Data governance.* Six articles [63,65–67,69,70]^.^emphasized the importance of establishing and strengthening robust governance frameworks, developing clear guidelines, and regulating data access on shared platforms, all of which significantly influence data sharing practices [70]^..^

#### 7. Barriers to the sharing of genomic data.

7.1 *Lack of trust.* Five articles [63,32,65] The findings revealed that lack of trust in those requesting access to the data, coupled with uncertainty about their intentions for its use, constituted key barriers to data sharing. Concerns were also raised about sharing genomic data with profit-driven companies and the associated risks of commercialization. Moreover, participants were hesitant to share their data due to previous instances of data misuse they had witnessed, as well as ongoing challenges surrounding data protection and safeguarding personal information [32,66].

7.2 Infrastructural challenges. One article [25] highlighted the infrastructural challenges that affect the sharing of genomic data such as research environments often lack adequate infrastructure, limited funding, and support, which limits the ability of researchers to participate fully in collaborative projects. Limited internet access, power outages, and outdated hardware/software hinder LMIC scientists’ ability to engage in data sharing and international collaborations effectively. These infrastructural challenges are barriers to the sharing of genomic data.

7.3 *Cultural and contextual differences*: One article indicated that LMIC scientists may perceive openness and data sharing differently due to their specific research contexts and infrastructural challenges. Initiatives that work in high income countries may not translate easily to LMIC settings that would require context-specific approaches [25].^.^

## Discussion

Our synthesis of the 11 included articles indicates that, despite the substantial benefits of genomic data sharing, there is a clear need for critical examination of the associated ethical, legal, and social issues (ELSI). This systematic review identifies key concerns, including the importance of trust in collaborative research given the sensitive nature of genomic data, the need to safeguard participant privacy and confidentiality, existing legal gaps in data protection, and social risks such as data misuse, stigmatization, and potential harm to individuals and communities. Overall, the review contributes to ongoing discussions on responsible and equitable genomic data sharing, with particular relevance for low- and middle-income country contexts.

The findings reveal that researchers share genomic data in both formal and informal ways. On the formal side, data is shared through institutional repositories and established data platforms. But many also rely on informal methods like emailing data to trusted colleagues or using cloud storage services. These informal practices seem to persist because they’re convenient and sometimes more accessible, especially in settings where formal systems are limited or slow. This reflects what others have found in sub-Saharan Africa [25,70], and it underscores the need for data sharing approaches that are not only ethical and secure, but also practical and grounded in how researchers actually work.

This approach is sometimes used as a strategy to protect the privacy and confidentiality of research participants and this is in agreement with Yilmaz et al. [72], who indicates that as much as possible sensitive data should not be shared. These findings align with views of The findings align with views of Oestreich et al., 2021 and Cheah et al., 2015 [73,74] which showed that shared data with meta data increases privacy risks making it easy to be re-identified.

This review demonstrates that trust is a critical component during the sharing of genomic data because of its sensitive nature with far reaching considerations beyond the individual. If researchers hold reservations or distrust regarding the intentions of individuals or entities seeking access to the data, it may manifest as a reluctance or unwillingness to share the participant genomic data. The findings are an indication that trust in the sharing of genomic data is critical which concurs with research conducted by Jackson et-al 2019 [66] on the role of trust and whole genome sequencing [63,65,66,75]. The findings on mistrust of researchers during the sharing of data aligns with a review by Amoakoh et al 2023 which showed that local researchers and participants sometimes exhibit a lot of trust in the collaborations but the partners sometimes are not respectful of the set terms and conditions [43].

The review highlights the ethical concern of limited access to shared data for researchers from LMICs, as evidenced in several articles. This prompts an inquiry: is this situation a result of researchers lacking skills or the necessary infrastructure, or does it stem from the diminished moral regard sometimes associated with researchers from the Global South? This could imply limited skill set and inadequate infrastructure of LMICs leading to over reliance on developed countries. Therefore, research institutions should have their capacities strengthened and these findings align with research which was conducted from Africa [67,76]. In addition, the unequal power dynamics, could create a situation where ownership and control of genomic data may primarily rest with institutions from high-income countries [65]. We draw attention to the need to decolonize research ethics by addressing issues of power imbalances, promoting fair and local control of data ownership, ensuring equitable benefit sharing, and developing robust regulatory frameworks [77,30]. This dynamic shapes important distinctions between data sharing and data transfer, as decision-making authority over access, use, and downstream benefits often rests with actors in better-resourced settings. As a result, what is framed as “data sharing” may, in practice, function as unilateral data transfer, reinforcing existing power asymmetries and limiting the ability of researchers and institutions in less-resourced contexts to exercise meaningful control over their data.

The ethical and social implications highlighted in this review include the risk of re-identifiability, challenges in the consenting process, fear of exploitation from previous historical experiences and inequity in collaborative research. Despite the H3Africa and the GA4GH technical safe guards with strict access controls and backup, genomic data remains vulnerable to the risk of re-identification [78,79]. The findings highlight how the risk of re-identifiability remains a major ethical concern in the sharing of genomic data, which comes along with breach of confidentiality, loss of privacy, and social implications. We reflect on whether genomic data can ever fully be de-identified even with the implementation of technical safeguards designed to reduce the risk and especially in situations where data protection systems are still evolving.

Our findings align with previous studies that highlight the persistent challenge of re-identifiability and privacy breaches, noting that existing safeguards cannot completely prevent the possible linkage of genomic data with demographic or other datasets.[22,23,24]. The ethical and social concerns identified in this review align with findings from studies on trust and privacy by Jackson et al. [66] and Oestreich et al. [74] [66,74]. Similar studies as well revealed that many publicly available genomic data files included personal identifiers such as individuals’ first and last names within file labels. This raised serious concerns about re-identifiability, which could expose individuals, families, and even communities to risks of shame, stigmatization, and labeling. Participants in these studies also expressed unease about the potential for data misuse [80,32,74]. However, the findings of this review are contrary to research by Goodman et al. 2017 [1] who surmised that the benefits of sharing data were far greater than the risks involved [1]. Our opinion on the issue of re-identifiability not being a point of concern differs from research by Goodman et al. 2017 [1] because a breach of confidentiality could expose research participants to the risks of stigmatization, shame, and labeling of the community. While our findings contrast with those of Byrd et al. [2], who reported that in some cases, genomic data poses minimal or no risk., they do align with the review's conclusion that the risk of re-identification increases when shared data accurately describes a person for long periods of time [2]. The review highlighted ethical concerns related to the use of shared data uploaded to international databases for commercial purposes. The findings suggest worries, particularly among researchers and communities from LMICs, about being excluded from the benefits of the data they contributed. Such data may be used to develop new diagnostics or therapeutics that are subsequently sold back to LMICs at high and often unaffordable prices. These findings align with a study conducted in Uganda that adopted a distributive justice lens, where the authors questioned the moral justification for commercializing genomic data freely contributed by participants, highlighting concerns that those who provide the data may not receive a fair or proportionate share of the resulting benefits [81]. The review is indicative that the inequity is heighted by the absence of benefit-sharing models in the current ethical and legal frameworks in LMICs which concurs with [65,67,68]. Benefit sharing frameworks are critical in the sharing of genomic data in collaborative relationships. These rooted disparities highlight the need to decolonize research ethics by promoting fair authorship, shared governance, and equitable participation in global health research [29,30]. This necessitates a rigorous analysis of decolonization frameworks to ensure equitable benefit-sharing and prevent the existing global health inequities, particularly as data sharing becomes a prerequisite for research funding [30].

The legal landscape surrounding genomic data sharing is complex with current laws and regulations lagging behind. Several LMICs have developed Data Protection Acts though with gaps. Staunton et al 2019 [82] emphasized a gap in the Acts in Africa and noted that while they are increasingly being introduced, there is still insufficient attention being given to the specific regulation of data sharing for genomic research [76,82]. Mweemba et al. [71] indicates that while the National Health Research Act of 2013 aims to protect Zambian investigators by requiring specific consent, there are concerns that the stringent regulatory environment could hinder genomic research [71]. In developing the guidelines and regulations, policy makers should be cognizant not to stifle the conduct of genomic research which is dependent on the sharing of genomic data. Clarifying ownership rights is crucial for establishing clear guidelines for data sharing and ensuring that individuals have control over their own genetic information as well as necessitating the harmonization of legal standards to facilitate data sharing while protecting the rights of individuals is critical. While guidelines have been developed, several local researchers may not be conversant with the guidelines and regulations that govern genomic data sharing and data protection. This may be attributed to the fact that the necessary guidelines are either lacking, outdated, or altogether nonexistent which concurs with [33]. Guidelines developed from elsewhere may not be in a position to adequately protect researchers and participants [74]. Our findings align with conclusions from a scoping review by 1 regarding the need for context specific guidelines and strengthening the legal system around the regulation of genomics and data sharing. The review indicated that despite the presence of genomic guidelines, they were not referred to [31]. This is crucial because genomic data is highly sensitive personal data, and the lack of clear regulations can raise ethical and privacy concerns.

Establishing robust governance frameworks that prioritize ethical considerations and protect individual rights is paramount. These frameworks should incorporate principles of transparency, accountability, and de-identification of data, data access, consent, respect of individuals, families and communities and sharing the benefits accrued from the research

### Strengths and limitations

This review offers valuable insights into the context, facilitators and barriers to the sharing of human genomic data sharing by leveraging qualitative evidence.

While it effectively highlights key factors such as data de-identification, responsible sharing practices, and the presence of data protection frameworks. A quality assessment was conducted of the studies which were included in the review which promotes the reliability and generalizability of the findings.

This review adds to the growing body of knowledge on the ELSI in genomic data sharing, highlighting the need for further inquiry to help protect the rights of both researchers in LMICs and research participants.

The findings of this review will guide regulators and policy makers in determining the best how to protect the personal interests of research participants while enabling the sharing of data through Global research networks.

Our reporting is aligned to the PRISMA statement [83,84] and the full report will be shared with relevant stakeholders including universities, civil society, funders, and departments of genomic and genetic research to ensure an adequate reach especially in LMICs. The high level of agreement (92%) demonstrates strong inter-rater reliability and suggests that the data extraction process was applied consistently.

The review conducted a comprehensive search of relevant papers up to the year 2025. We synthesized data from a wide range of databases which provides data from a relevant scope [43]. This ensures that the findings are based on the most recent and relevant literature available. This context is crucial for understanding the regulatory environment that influences data sharing practices.

A limitation of our review is its primary focus on qualitative studies, which meant that only a small number of quantitative articles were included which may affect generalizability. However, we carefully examined their methodologies to ensure they demonstrated sufficient rigour before incorporating their findings. In addition, the review concentrated on studies conducted in LMICs, with a particular emphasis on African contexts, which may limit the generalizability of the findings to other regions. The use of a narrative synthesis approach also presents limitations, as it relies heavily on reviewer judgement when interpreting and organizing themes, introducing the potential for subjective interpretation and bias. Finally, we did not conduct a meta-analysis due to the heterogeneity of study designs and outcomes.

Overall, our comprehensive approach in reviewing only a few articles as well as inclusion of recent literature provides an indepth understanding of the context and ethical, legal, and practical considerations in the sharing of human genomic data. This approach enabled us to engage critically with each study, identify indirect but important themes, and generate a richer, more context-sensitive synthesis than would have been possible with a broader but less detailed review.

## Conclusion

While the sharing of genomic data holds great promise for advancing biomedical, it is accompanied by significant ethical, social, and legal challenges that must be carefully navigated. This systematic review highlights the importance of informed consent, equity, trust-building, and strong legal protections in the context of genomic data utilization. Based on an analysis of various articles, we assert that it is feasible to leverage the benefits of genomic data sharing while safeguarding the rights and interests of individuals and communities. Achieving this balance necessitates the establishment of ethical governance frameworks that facilitate data accessibility while ensuring the protection of participants.

## Recommendations

Policymakers and research institutions should actively strengthen protections for research participants’ privacy by mandating enhanced consent processes that explicitly explain potential future uses of genomic data, cross-border data sharing arrangements, and associated risks and benefits. Research ethics committees should require evidence that consent materials are written in accessible language and that consent discussions include opportunities for participants to ask questions and revisit decisions over time.

National regulators and policy makers should develop and operationalize ethical and regulatory frameworks that include enforceable provisions on genomic data governance. These frameworks should specify conditions for data access and secondary use, require fair and transparent data access agreements, and clearly outline benefit-sharing obligations. Such obligations should include co-authorship for LMIC researchers, structured capacity-strengthening commitments, and shared governance mechanisms that enable LMIC institutions to participate meaningfully in data-related decision-making.

Research institutions should implement mechanisms to safeguard the interests of LMIC researchers by standardizing data sharing agreements that promote equitable partnerships. These mechanisms should ensure that LMIC researchers are involved in decisions regarding data transfer, access approvals, and downstream use, rather than being limited to data collection roles.

Regulatory authorities and research institutions should increase public awareness of data protection laws and policies through structured community engagement. This should include developing simplified policy summaries, conducting regular community dialogues, and systematically engaging community advisory boards to support ongoing understanding of genomic research and data sharing practices.

Institutions in LMICs, in collaboration with funders and international partners, should invest in targeted capacity-building programs. These programs should focus on strengthening technical skills in sequencing and data analysis, as well as negotiation, contract management, and governance competencies, to enable LMIC researchers to participate as equal partners in advanced genomic research and international collaborations.

### Reporting and dissemination

Our reporting follows the PRISMA statement guidelines [83,84], which emphasize transparency and openness in research and are aligned with the principles of the Open Science Framework [85]. A policy engagement meeting is underway, and the full report will be disseminated to key stakeholders, including universities, civil society organizations, funders and departments responsible for genomic research. This will also involve community engagement to ensure broader public understanding and participation. In addition, preliminary results from the review have been disseminated at both local and international conferences. Together, this dissemination strategy is intended to ensure that the findings reach relevant decision makers and contribute to ongoing policy development and practice in LMIC settings.

### Appendix

An assessment of the methodological quality of the included studies is presented in Table 4.

**Table 4:** Quality assessment of the included studies (*n* = 10).

Author, year	Is the aim of the study indicated?	Is the research design appropriate?	Was ethical approval reported?	Was the sampling strategy indicated?	Was the sample size indicated?	Was data analysis sufficiently rigorous?	Are the findings adequately derived from the data?	Was the influence of the researcher on the research addressed and vice-versa	Were the voices of the participants captured?	Is there coherence between the data sources, collection, analysis and interpretation?	Rating	Score
Mafigiri,2023	Y	Y	Y	Y	Y	Y	Y	N	Y	Y	9	Good
deVries,2014	Y	N	Y	N	Y	Y	Y	N	Y	Y	8	Good
[66]	Y	N	Y	Y	Y	Y	Y	N	Y	Y	8	Good
[65]	Y	Y	Y	Y	Y	Y	Y	Y	Y	Y	9.5	Good
[68]	Y	N	Y	Y	Y	Y	Y	N	Y	Y	8	Good
[71]	Y	Y	Y	Y	Y	Y	Y	N	Y	Y	8	Good
Cengiz [64]	Y	Y	Y	Y	Y	Y	Y	N	Y	Y	9	Good
Brown [63]	Y	N	Y	Y	Y	Y	Y	N	Y	Y	8	Good
Kabanda [70]	Y	Y	Y	Y	Y	Y	Y	N	Y	Y	9	Good
Kofi (2025)	Y	N	Y	Y	Y	Y	Y	N	Y	Y	8	Good

Scores of **8–10 (Good), 5–7 (Fair), 0–4 (Poor)**

## Supporting information

S1 Checklist populated. (DOCX)

S2 File Search strategy. (DOC)

## Abbreviations

ELSI: Ethical, legal and social implications
JBI: Joanna Briggs Institute
LMICs: Low and middle income countries
NR: Not reported
PICOST: Population intervention context outcome setting and time frame
PRISMA: Preferred Reporting Items for Systematic Reviews and Meta-Analyses
