# Supplementary material for: Context of data sharing practices in collaborative human genomic research in low and middle income countries: A systematic review
Source: PLoS One. 2026 Aug 7;21(8):e0354471. doi: 10.1371/journal.pone.0354471 (PMC13450839; doi:10.1371/journal.pone.0354471)
Supplement: Supplementary file 2 [file pone.0354471.s002.doc]

**Appendix 2: Search string**

| No. | Search strategy | Data base |
| --- | --- | --- |
| 1. | Genomics or genetic or genetics or human genome or human genomics  AND  Data or data sharing or data link or data sharing practices or data breach or data re-use  AND  (Angola[tw] OR Benin[tw] OR Botswana[tw] OR Burkina Faso[tw] OR Burundi[tw] OR Cameroon[tw] OR Canary Islands[tw] OR Cape Verde[tw] OR Central African Republic[ tw] OR Chad[tw] OR Comoros[tw] OR Congo[tw] OR Democratic Republic of Congo[tw] OR Djibouti[tw] OR Egypt[tw] OR Equatorial Guinea[tw] OR Eritrea[tw] OR Ethiopia[tw] OR Gabon[tw] OR Gambia[tw] OR Ghana[tw] OR Guinea[tw] OR Guinea Bissau[tw] OR Ivory Coast[tw] OR Cote d’Ivoire[tw] OR Jamahiriya[tw] OR Jamahiriya[tw] OR Kenya[tw] OR Lesotho[tw] OR Liberia[tw] OR Libya[tw] OR Libya[ tw] OR Madagascar[tw] OR Malawi[tw] OR Mali[tw] OR Mauritania[tw] OR Mauritius[tw] OR Mayotte[tw] OR Mozambique[tw] OR Mozambique[tw] OR Namibia[tw] OR Niger[tw] OR Nigeria[tw] OR Sao Tome and Principe[tw] OR Reunion[tw] OR Rwanda[tw] OR Senegal[tw] OR Seychelles[tw] OR Sierra Leone[tw] OR Somalia[tw] OR South Africa[ tw] OR Sudan[tw] OR Sudan Sudan OR Swaziland[tw] OR Tanzania[tw] OR Togo[tw] OR Uganda[tw] OR Western Sahara[ tw] OR Zaire[tw] OR Zambia[tw] OR Zimbabwe[ tw] OR Central Africa[tw] OR Central African[tw] OR West Africa[tw] OR West African[tw] OR Western Africa[tw] OR Western African[tw] OR East Africa[tw] OR East African[tw] OR Eastern Africa[tw] OR Eastern African[tw] OR South African[ tw] OR Southern Africa[tw] OR Southern African[tw] OR sub Saharan Africa[tw] OR sub Saharan African[tw] OR sub-Saharan Africa[tw] OR sub-Saharan African[tw]) NOT (guinea pig[tw] OR guinea pigs[tw] OR aspergillums Niger[tw]) | Pubmed |
| 2. Revised search | The following terms were combined to search the PubMed database as follows:  (Researchers OR regulators OR participants) AND (Genomics OR genetic OR genetics OR human genome OR human genomics) AND (Data or data sharing or data link OR data sharing practices OR data breach OR data re-use) AND (low and middle income countries OR low and middle income country OR middle income country OR middle income population OR under developed country, OR lower income nations OR lower income populations OR underserved countries OR LMIC OR LMICs OR third world OR underserved population OR deprived country OR deprived population OR deprived populations OR poor country OR poor countries OR poor nation OR poor nations OR poor population OR poor populations OR poor world OR poorer countries OR poorer nations OR poorer population OR poorer populations OR developing economy OR developing economies OR less developed economy OR less developed economies OR underdeveloped economies OR middle income economy OR middle income economies OR low income economy OR low income economies OR lower income economies OR low gdp OR low gnp OR low gross domestic OR low gross national OR lower gdp OR lower gross domestic OR lami country OR lami countries OR transitional country OR transitional countries OR emerging economies OR emerging nation OR emerging nations). | Pubmed |
|  |  | 594 |
|  |  |  |
| 3. | TI=(Genomics OR genetic OR genetics OR human genome OR human genomics)  AND  TI=(Data or data sharing or data link or data sharing practices or data breach or data re-use)  AND  TI=(Angola OR Benin OR Botswana OR Burkina Faso OR Burundi OR Cameroon OR Canary Islands OR Cape Verde OR Central African Republic OR Chad OR Comoros OR Congo OR Democratic Republic of Congo OR Djibouti OR Egypt OR Equatorial Guinea OR Eritrea OR Ethiopia OR Gabon OR Gambia OR Ghana OR Guinea OR Guinea Bissau OR Ivory Coast OR Cote d’Ivoire OR jamahiriyah OR Kenya OR Lesotho OR Liberia OR Libya OR Libya OR Madagascar OR Malawi OR Mali OR Mauritania OR Mauritius OR Mayotte OR Mozambique OR Mozambique OR Namibia OR Niger OR Nigeria OR Sao Tome and Principe OR Reunion OR Rwanda OR Senegal OR Seychelles OR Sierra Leone OR Somalia OR South Africa OR Sudan OR South Sudan OR Swaziland OR Tanzania OR Togo OR Uganda OR Western Sahara OR Zaire OR Zambia OR Zimbabwe OR Central Africa OR Central African OR West Africa OR West African OR Western Africa OR Western African OR East Africa OR East African OR Eastern Africa OR Eastern African OR South African OR Southern Africa OR Southern African OR sub Saharan Africa OR sub Saharan African OR sub-Saharan Africa OR sub-Saharan African OR aspergillus Niger) | Web of Science |
| REVISED SEARCH-10 JUNE2022-2025 |  |  |
| 2003-2025 |  | 340 |
|  |  |  |
| 4. | (Genomics OR genetic OR genetics OR human genome OR human genomics) AND (Data or data sharing or data link or data sharing practices or data breach or data re-use) AND (sub Saharan Africa OR sub-Saharan Africa) | Google scholar Search strategy |
| 2003-2025 |  | 1127 |
|  |  |  |
|  | TARGETED SOURCES | 30 |
|  | TOTAL RETRIEVED | 2091 |
|  | DUPLICATES | 650 |
|  | **FOR SCREENING** | **1,441** |
